# Face and content validity of an instrument to measure dampness and mold damage in a Spanish-speaking Latin American context

**DOI:** 10.15649/cuidarte.4130

**Published:** 2024-11-15

**Authors:** Raquel Rivera-Carvajal, Diana Carolina Tiga-Loza, D. Jimena Roncancio, Laura Andrea Rodriguez-Villamizar, Miguel José Galván-Ramírez, Edgar David Gómez-Gómez, Adriana Ximena Sandoval-Meza, Beatriz Elena Guerra-Sierra

**Affiliations:** 1 Universidad de Santander, Facultad de Ciencias Médicas y de la Salud, Instituto de Investigación Masira, Bucaramanga, Colombia. E-mail: raq.rivera@mail.udes.edu.co Universidad de Santander Universidad de Santander Facultad de Ciencias Médicas y de la Salud Instituto de Investigación Masira Bucaramanga Colombia raq.rivera@mail.udes.edu.co; 2 Universidad de Santander, Facultad de Ciencias Médicas y de la Salud, Instituto de Investigación Masira, Bucaramanga, Colombia. E-mail: dia.tiga@mail.udes.edu.co Universidad de Santander Universidad de Santander Facultad de Ciencias Médicas y de la Salud Instituto de Investigación Masira Bucaramanga Colombia dia.tiga@mail.udes.edu.co; 3 Universidad el Bosque, Grupo Agua, Salud y Ambiente. Bogotá, Colombia. E-mail: djroncancio@unbosque.edu.co Universidad El Bosque Universidad el Bosque Grupo Agua, Salud y Ambiente Bogotá Colombia djroncancio@unbosque.edu.co; 4 Universidad Industrial de Santander, Escuela de Medicina, Bucaramanga, Colombia. E-mail: laurovi@uis.edu.co Universidad Industrial de Santander Universidad Industrial de Santander Escuela de Medicina Bucaramanga Colombia laurovi@uis.edu.co; 5 Universidad de Santander, Facultad de Ciencias Médicas y de la Salud, Instituto de Investigación Masira, Bucaramanga, Colombia. E-mail: migalramirez874@gmail.com Universidad de Santander Universidad de Santander Facultad de Ciencias Médicas y de la Salud Instituto de Investigación Masira Bucaramanga Colombia migalramirez874@gmail.com; 6 Universidad de Santander, Facultad de Ciencias Médicas y de la Salud, Instituto de Investigación Masira, Bucaramanga, Colombia. E-mail: edavidgomez17@gmail.com Universidad de Santander Universidad de Santander Facultad de Ciencias Médicas y de la Salud Instituto de Investigación Masira Bucaramanga Colombia edavidgomez17@gmail.com; 7 Universidad de Santander. Facultad de Ciencias Exactas Naturales y Agropecuarias. Grupo Microbiota, Bucaramanga, Colombia. E-mail: asandoval@udes.edu.co Universidad de Santander Universidad de Santander Facultad de Ciencias Exactas Naturales y Agropecuarias Grupo Microbiota Bucaramanga Colombia asandoval@udes.edu.co; 8 Universidad de Santander. Facultad de Ciencias Exactas Naturales y Agropecuarias. Grupo Microbiota, Bucaramanga, Colombia. E-mail: bguerra@udes.edu.co Universidad de Santander Universidad de Santander Facultad de Ciencias Exactas Naturales y Agropecuarias Grupo Microbiota Bucaramanga Colombia bguerra@udes.edu.co

**Keywords:** Fungi, Humidity, Home Health Nursing, Indoor Environment, Home Visit, Indoor Air Pollution, Hongos, Humedad, Atención Domiciliaria de Salud, Ambiente en el Hogar, Visita Domiciliaria, Contaminación del Aire Interior, Fungos, Umidade, Assistência Domiciliar, Ambiente Domiciliar, Visita Domiciliar, Poluição do Ar em Ambientes Fechados

## Abstract

**Introduction:**

Exposure to dampness and mold in houses can lead to health problems among residents.

**Objective:**

To assess the face and content validity of the "Dampness and Mold Assessment Tool. General Buildings" instrument, proposed by the Centers for Disease Control and Prevention - National Institute for Occupational Safety and Health, for use in Spanish-speaking Latin American contexts.

**Materials and Methods:**

A face and content validation study was conducted through expert evaluation using the Delphi method. The Content Validity Index (CVI) was calculated for clarity, coherence, sufficiency, and relevance, as well as the level of agreement among raters.

**Results:**

A total of 20 expert evaluators participated, with an average of 18.5 ± 9.09 years of experience; 50% held doctoral degrees, and the other 50% held master’s degrees. The CVI scores were above 0.75 for all items, except for "room/area type" which had a CVI of 0.65. Agreement among experts was statistically significant (p < 0.05) except for "room/area type" (p = 0.055). Adjustments to the instrument were made based on the evaluators' recommendations.

**Discussion:**

This study is one of the first to validate this instrument, with potential for adaptation to various settings beyond residential, including hospitals, educational institutions, and workplaces.

**Conclusion:**

The face and content validation process enabled the development of an instrument for assessing dampness and mold damage in Spanish-speaking Latin American settings, generating a semi-quantitative indicator. This tool is recommended for use in home visits and research to support data on factor validity, Rasch analysis, and reliability in its application.

## Resumen.

**Introducción:**

La exposición a humedad y mohos en las viviendas pueden generar afectaciones en la salud de las personas que las habitan.

**Objetivo:**

Estimar la validez facial y de contenido de la traducción del instrumento "*Dampness and Mold Assessment Tool. General Buildings" propuesto por el Centers for Disease Control and Prevention -National Institute for Occupational Safety and Health* para uso en el contexto Latinoamericano hispanohablante.

**Materiales y Métodos:**

Estudio de validación facial y de contenido mediante evaluación de expertos, con el método Delphi. Se estimó Índice de Validez de Contenido (IVC) para claridad, coherencia, suficiencia y relevancia, y concordancia entre los jueces.

**Resultados:**

20 jueces expertos participaron el proceso, una experiencia promedio de 18.5 ± 9.09 años, el 50% con doctorado y 50% con maestría. Los puntajes de IVC fueron mayores a 0.75 en todos los ítems, excepto en el ítem “Tipo de cuarto/habitación/zona” con IVC=0.65, la concordancia fue significativa (p<0.05) excepto en “Tipo de cuarto/habitación/zona” valor p=0.055. Se realizaron ajustes al instrumento según recomendaciones.

**Discusión:**

El presente estudio es uno de los primeros relacionados con validación del instrumento, el tipo de zona puede ser adaptado según el área a evaluar, para este caso se adaptó a viviendas, pero se puede considerar a otras instituciones como hospitales, instituciones educativas, empresas u otras.

**Conclusión:**

El proceso de validación facial y de contenido permite obtener un instrumento para usar en el contexto latinoamericano hispanohablante con el objeto de determinar afectaciones por humedad y mohos a nivel locativo que genera un indicador semi-cuantitativo, se invita a considerar su uso en procesos de visitas domiciliarias, en investigaciones que posteriormente favorezcan datos de validez de factores, análisis Rasch y concordancia en el uso del instrumento.

## Resumo.

**Introdução:**

A exposição à umidade e ao mofo nas residências pode afetar a saúde das pessoas que ali residem.

**Objetivo:**

Estimar a validade facial e de conteúdo da tradução do instrumento "Ferramenta de Avaliação de Umidade e Molde. Edifícios Gerais" proposto pelo Centers for Disease Control and Prevention -National Institute for Occupational Safety and Health para uso na América Latina de língua espanhola. contexto.

**Materiais e Métodos:**

Estudo de validação facial e de conteúdo por meio de avaliação de especialistas, com método Delphi. Estimou-se o Índice de Validade de Conteúdo (IVC) quanto à clareza, coerência, suficiência e relevância, e concordância entre os juízes.

**Resultados:**

Participaram do processo 20 juízes especialistas, experiência média de 18,5 ± 9,09 anos, sendo 50% com doutorado e 50% com mestrado. As pontuações do IVC foram superiores a 0,75 em todos os itens, exceto no item “Tipo de cômodo/quarto/zona” com IVC=0,65, a concordância foi significativa (p<0,05) exceto no item “Tipo de cômodo/quarto/zona”. Valor de p = 0,055. Foram feitos ajustes no instrumento conforme recomendações.

**Discussão:**

O presente estudo é um dos primeiros relacionados à validação do instrumento, o tipo de área pode ser adaptado de acordo com a área a ser avaliada, neste caso foi adaptado para residências, mas outras instituições podem ser consideradas como hospitais, instituições de ensino, empresas ou outros.

**Conclusão:**

O processo de validação facial e de conteúdo permite obter um instrumento a ser utilizado no contexto latino-americano de língua espanhola para determinar os efeitos devidos à umidade e ao mofo em nível local que gera um indicador semiquantitativo, convida se a considerar sua utilização em processos de visitas domiciliares, em pesquisas que posteriormente favorecem dados de validade fatorial, análise Rasch e concordância na utilização do instrumento.

## Introduction

The prevalence of dampness and mold in houses has been estimated to range between 10% and 50% of residences^1^. This exposure is associated with allergic respiratory diseases, such as asthma exacerbation, allergic rhinitis, and bronchitis^2^. However, evidence linking other conditions, like chronic obstructive pulmonary disease (COPD), dermatological issues, rheumatic disorders, arthritis, cancer, and neurotoxic effects, remains limited^3^. Research indicates that the severity of structural damage in houses is correlated with increased respiratory symptoms. For asthma events, approximately 20% (95% CI: 12-29%) of cases are attributable to indoor mold exposure, generating annual costs estimated at USD 3.5 billion^4^. Mold exposure has also been linked to the increased severity of symptoms associated with Sick Building Syndrome^5^. Furthermore, climate change particularly the increase in indoor temperatures can promote conditions conducive to dampness and mold growth in houses^6^.

For health professionals, especially those in nursing who conduct houses visits, it is crucial to identify environmental risks within residences^7^ and provide recommendations to reduce exposure that could lead to the development or exacerbation of health conditions, particularly in individuals with a history of asthma^8^^, ^^9^. Adequate ventilation and minimizing mold exposure are key preventive strategies for these populations.

Various tools have been utilized to assess dampness and mold damage, such as the Subjective Indoor Air Quality (SIAQ) tool^10^, The MM questionnaries^11^, ASHRAE (American Society of Heating, Refrigerating, and Air Conditioning Engineers, Atlanta) ^12^, visual assessments^13^, and measurements based on affected areas in square centimeters, humidity odors, paint and wallpaper damage, among others^14^. These tools generate a variety of qualitative and semi-quantitative measurements, which complicates comparisons across affected areas.

To address the variability in indicators used to assess dampness and mold damage, the National Institute for Occupational Safety and Health (NIOSH) at the Centers for Disease Control and Prevention (CDC) developed an instrument that consolidates the mentioned characteristics, generating a semi-quantitative indicator^15^. This tool evaluates various components present in a room or area, such as walls, ceilings, floors, windows, furnishings, ventilation systems, materials, pipes, and any other necessary elements. It assigns a score based on mold odors, stains, visible mold, and wetness, subsequently producing an overall semi-quantitative score for each room or area. It is worth noting that the CDC-NIOSH has made the instrument and an Excel sheet for tabulating and obtaining the indicator freely available for public use^16^.

Given the need for a standardized instrument, the present study aimed to validate the face and content of the "Dampness and Mold Assessment Tool. General Buildings" (DMAT) translation for application in Spanish-speaking Latin American contexts.

## Materials and Methods

### Design

A face and content validation study was conducted for the DMAT instrument, including translation, back-translation, cross-cultural adaptation, and expert evaluation. The validation process took place between February and April 2024, using the Delphi technique^17^.

### Instrument description

An online version of the instrument was created in Google Forms, providing links to both the original (English) version and the adapted Spanish version. The form included informed consent, expert characterization (name, gender, age, nationality, residence, highest level of education, and professional experience). For face validity, clarity and coherence were assessed, while content validity was evaluated based on sufficiency and relevance for each component of the DMAT instrument. A Likert scale from 1 to 5 (1: the item does not meet the criterion at all; 5: the item fully meets the criterion) was used.

### Expert rater selection criteria

Experts were selected based on a convenience sampling approach. The inclusion criteria required professional experience in fields such as research, public health, epidemiology, civil engineering, environmental health, environmental health policy, occupational health, mycology, or instrument validation; experience as an expert rater in at least one validation study; and a minimum academic qualification of a master’s or doctoral degree. Exclusion criteria included having less than one year of professional experience in the relevant field.

### Data analysis

Characteristics of the raters were described using relative and absolute frequencies for categorical variables and means and standard deviations for numerical variables. Normal distribution of data was verified using the Shapiro-Francia test. The Content Validity Index (CVI) was calculated, responses rated in categories 4 and 5, deemed acceptable, and was divided by the number of experts for each criterion: clarity, coherence, sufficiency, and relevance. A CVI score above 0.75 was considered desirable. For each item and its components, the mean and standard deviation of scores assigned by the raters were calculated. Agreement among experts was evaluated using Brennan and Prediger's kappa statistic^18^, with agreement levels interpreted as follows: poor (<0.00), low (0.00-0.20), fair (0.21-0.40), moderate (0.41-0.60), substantial (0.61-0.80), and almost perfect (0.81-1.00). Stata version 17 and Excel were used for data analysis. The data generated in this study, as well as the final validated instrument version, are available on Mendeley Data^19^.

### Ethical considerations

The Bioethics Institutional Review Board -IRB at Universidad de Santander provided approval for the study as part of the project “Indoor Environmental Molds and Mild Cognitive Impairment in Older Adults in Bucaramanga” (Minutes No. 01, February 20, 2024). The study adhered to ethical principles of autonomy, beneficence, and non-maleficence.

## Results

### Characteristics of the expert raters

A panel of 20 expert raters was assembled, with a higher percentage of female participants and an average age of 45 years. The raters were from Colombia, Mexico, Peru, Spain, and Venezuela, with 50% holding master’s degrees and the other 50% holding doctoral degrees. Their areas of expertise included public health, environmental health, engineering, occupational health, and mycology, with an average of 18 years of professional experience across research, teaching, and independent consulting (see Table 1).

**Table 1:** Characteristics of expert raters

Variable	%(n) (20)
Sex	
Female	65.00(13)
Male	35.00(7)
Mean age ± SD	45.05 ± 10.44
Country	
Colombia	70.00(14)
Mexico	10.00(2)
Peru	10.00(2)
Spain	5.00(1)
Venezuela	5.00(1)
Education level	
Masters	50.00(10)
Doctorate	50.00(10)
Areas of expertise	
Epidemiology	40.00(8)
Environment	45.00(9)
Public health	50.00(10)
Occupational health	25.00(5)
Mycology	5.00(1)
Engineering	35.00(7)
Mean years of experience ± SD	18.5 ± 9.09
Working experience	
Researcher	85.00(17)
Professor	80.00(16)
Independent consultant	40.00(8)

The Content Validity Index (CVI) scores for each item and criterion, along with agreement levels among experts, are presented in Table 2. The following is a summary of findings for each item:

General Information Item: CVI scores for clarity, coherence, sufficiency, and relevance were above 0.75, with mean ratings exceeding 4. Agreement had a coefficient of 0.15 (95% CI: 0.06; 0.24), p < 0.05. Experts suggested adding details on "neighborhood and city" under the housing type, which were incorporated into the instrument.

Room/Area Type Item: The CVI for sufficiency was 0.65, with a mean rating of 3.65 ± 1.18, while clarity had a mean score below 4. The agreement showed a coefficient of 0.15 (95% CI: -0.006; 0.31), with p = 0.055. Expert raters recommended replacing "type of room" with "room" and including other options such as library and service room and for non-residential spaces or areas like gym, hallway, classroom, and office. These modifications were added, with types of room now including bedroom, kitchen, living room, dining room, living/dining room, bathrooms, study room, and "other (specify)."

Mold Odor Item: CVI scores were above 0.84, with mean ratings above 4.1. Agreement among raters showed a coefficient of 0.19 (95% CI: 0.15; 0.24), p < 0.001. Expert raters suggested clarifying how to differentiate mold odor from other smells, adding an option for "unknown source," and renaming categories to "none, mild, moderate, intense" with descriptions for each intensity level.

Room/Area Components Item: CVI scores were 0.80 or higher, with mean ratings exceeding 4. Agreement had a coefficient of 0.21 (95% CI: 0.11; 0.30), p < 0.001. Expert raters recommended adding “clothing” as an item to assess.

**Table 2:** CVI and mean scores for DMAT Items

Item	Clarity		Coherence		Sufficiency		Relevance		Agreement	
	CVI	X ± SD	CVI	X ± SD	CVI	X ± SD	CVI	X ± SD	Coeff (IC 95%)	P-value
General information	0.8	4.05 ± 0.82	0.8	4.15 ± 0.87	0.75	4 ± 1.12	0.9	4.25 ± 0.91	0.15(0.06; 0.24)	0.012
Room/area type	0.8	3.95 ± 0.75	0.75	4.05 ± 0.99	0.65	3.65 ± 1.18	0.9	4.35 ± 0.81	0.15(-0.006; 0.31)	0.055
Mold odor	0.9	4.25 ± 0.78	0.85	4.15 ± 1.08	0.85	4.2 ± 0.95	0.85	4.35 ± 0.87	0.19(0.15; 0.24)	0.001
Room/area components	0.9	4.25 ± 0.91	0.8	4.2 ± 0.89	0.85	4.05 ± 1.05	0.9	4.45 ± 0.82	0.21(0.11; 0.30)	0.006
Assessing damage and scoring	0.75	3.9 ± 0.91	0.9	4.4 ± 0.82	0.8	4.2 ± 0.83	0.95	4.55 ± 0.75	0.23(0.07; 0.38)	0.018
Components and assessment notes	0.75	4 ± 0.85	0.85	4.3 ± 0.86	0.95	4.35 ± 0.74	0.9	4.4 ± 0.82	0.21(0.10; 0.32)	0.009
Annexes	0.89	4.26 ± 0.80	0.85	4.4 ± 0.88	0.8	4.2 ± 0.89	0.75	4.05 ± 0.99	0.17(0.06; 0.29)	0.015

*p-value for Brennan and Prediger's Kappa Agreement Test*

Assessing Damage and Scoring Item: CVI scores were 0.75 or higher, with clarity ratings below 4 on average. Agreement had a coefficient of 0.23 (95% CI: 0.07; 0.38), p < 0.05. Experts suggested providing measurements in centimeters for better standardization.

Components and Assessment Notes: This item scored a CVI of 0.78 or higher, with average ratings above 4. Agreement had a coefficient of 0.21 (95% CI: 0.10; 0.32), p < 0.01. Raters suggested specifying clothing types (e.g., bedding, clothing, or cleaning items) and indicators (e.g., black or gray stains, signs of dampness).

Annexes: The annexes scored CVI values of 0.75 or higher, with mean ratings of 4 or above. Agreement among experts had a coefficient of 0.17 (95% CI: 0.06; 0.29), p < 0.05. Expert raters suggested using terms like “room” instead of “chamber” and specifying “door within 1 meter of an exterior entrance.” These adjustments were made accordingly Table 3.

**Table 3:** Experts' comments and research team responses

Comment	Research team responses
"The tool should lead to a general assessment and, at the end, indicate recommended actions to take, for example: medium risk - make improvements and reassess; high risk - this space should not be inhabited, etc."	We will consider incorporating this feedback after obtaining study data, to facilitate the generation of relevant categories.
"I recommend defining the profile of the instrument’s user."	The instrument is currently intended for trained personnel, such as inspectors; however, it should be noted that the instrument's authors have made it freely accessible for any potential users.
"Better characterization with area measurements (m²) and indicating if there are ventilation sources, and how many."	These important variables have been integrated into a separate instrument.
"I suggest explaining in Spanish what the acronym NIOSH means on the context page. Additionally, correct the wording in the fourth paragraph: ... that allows 'the' prioritize?..."	The indicated adjustments have been made.
"Add a glossary of terms"	The original document did not include specific terms, so the following were added: efflorescence, condensation, and appliances.

### Instrument adjustments based on expert feedback

Following CVI scores, mean ratings, and expert recommendations, adjustments were made to the instrument. The final version of the validated instrument is available on Mendeley Data^19^.

## Discussion

This research enabled the face and content validation of an instrument for assessing dampness and mold-related structural issues in the Latin American, Spanish-speaking context. The instrument's significant contribution lies in generating a semi-quantitative indicator, allowing comparisons across different areas and buildings, as well as over time as building improvements are made.

Previous studies have developed indices to quantify dampness and mold presence^10^^, ^^20^, but the variety of assessment tools has limited comparability. Therefore, this validation strongly supports the adoption of an instrument that integrates multiple components relevant to environmental health assessment. It is essential to recognize that dampness and mold exposure assessments can be conducted by trained professionals or through self-administered questionnaires. Proper training can enhance the accuracy of reports, and concordance or agreement evaluations can help ensure reproducibility when using the instrument.

One of the strengths of this study is the involvement of a substantial number of expert raters from various fields and Latin American countries. Additionally, the use of Brennan and Prediger’s kappa test to correct for chance agreement provides a reliable measure of inter-rater reliability, suitable for any number of raters and categorical ratings^21^. Given that laboratory identification of molds is often costly and time-consuming, using environmental site assessment as a practical approximation is a viable option^14^.

Limitations of this study include the instrument's lack of certain environmental factors that exacerbate mold growth, such as relative humidity, which favors mold growth in carpets^22^, construction materials^23^, the presence of volatile organic compounds, and ventilation adequacy5.

Standardizing methods for detecting dampness and mold damage is crucial, especially for monitoring environmental improvements aimed at reducing respiratory symptoms among occupants^24^. Future studies could explore factor analysis, Rasch analysis, and score concordance on agreement ratings to further validate the DMAT instrument.

For nurses conducting home visits, the instrument could be highly valuable in assessing household dampness and mold conditions in residential environments. This tool enables nurses to assess environmental factors in the homes and community spaces under their care^25^.

It is also worth noting that this instrument can be adapted to assess various types of environments, such as hospital settings, where structural issues related to dampness, mold, plumbing, and sewage must meet rigorous indoor environmental standards to ensure the safety of patients, particularly those with compromised immune systems.

## Conclusion

The face and content validation of the "Dampness and Mold Assessment Tool. General Buildings" (DMAT) by expert raters for Spanish-speaking Latin American contexts represents a significant advancement, facilitating its practical application across this region. Notably, the instrument provides a semi-quantitative indicator, enabling meaningful comparisons across various areas and facilitating monitoring changes following structural improvements. This tool is suitable for use in residential settings, including houses and apartments, and can be adapted to assess hospitals, workplaces, schools, universities, and other buildings seeking to quantifying dampness and mold-related damage to promote healthier indoor environments where people spend most of their time.
